# Serum metabolomic profiles associated with psychoneurological symptoms in women with early-stage breast cancer over one year

**DOI:** 10.3389/fonc.2026.1779012

**Published:** 2026-03-12

**Authors:** Gee Su Yang, Angela Starkweather, Tuo Lin, Tara Hashemian, Timothy J. Garrett, Dany Fanfan, Lakeshia Cousin, Shreya Patel, Debra Lynch Kelly, Debra E. Lyon

**Affiliations:** 1 School of Nursing, University of Connecticut, Storrs, CT, United States; 2 Division of Nursing Science, School of Nursing, Rutgers University, New Brunswick, NJ, United States; 3 Department of Biostatistics, University of Florida & Division of Quantitative Sciences, University of Florida Health Cancer Institute, Gainesville, FL, United States; 4 Division of Quantitative Sciences, University of Florida Health Cancer Institute, Gainesville, FL, United States; 5 Department of Pathology, Immunology and Laboratory Medicine, College of Medicine, University of Florida, Gainesville, FL, United States; 6 Biobehavioral Nursing Science, College of Nursing, University of Florida, Gainesville, FL, United States; 7 Tampa General Hospital, Tampa, FL, United States; 8 School of Medicine, University of Connecticut, Farmington, CT, United States; 9 Loewenberg College of Nursing, The University of Memphis, Memphis, TN, United States

**Keywords:** breast cancer, cancer survivorship, metabolite, psychoneurologic symptoms, race, chemotherapy

## Abstract

**Background:**

Breast cancer survivors frequently experience psychoneurological symptoms (PNS), such as pain, fatigue, anxiety, depression, and sleep disturbances, that persist beyond treatment and impair quality of life. Inflammatory and metabolic dysregulation, including alterations in the tryptophan/kynurenine pathway, have been implicated, yet longitudinal data and racial differences remain understudied. This study examined the longitudinal association between metabolite levels and PNS severity over time and explored their interactions with race.

**Methods:**

In a one-year longitudinal secondary data analysis, we performed untargeted serum metabolomic profiling and applied generalized estimating equations (GEE), adjusting for demographic covariates. Interaction terms were included to evaluate race-specific metabolite associations. Metabolite set enrichment analysis was conducted to identify impacted metabolic super-pathways using MetaboAnalyst 6.0.

**Results:**

Among 74 participants, we identified 140 metabolites significantly associated with PNS out of the 2,395 metabolites tested, with 38 named metabolites. Anxiety was associated with 2-aceto-2-hydroxy-butanoate (β=-2.40, p=5.79×10-6) and 1-pyrrolidinecarboxaldehyde (β=-0.753, p=8.61×10-6), while sleep disturbance associated with 4,6-O-ethylidene-D-glucose (β=9.83, p=4.82×10-6) and 5-hydroxytryptophol (β=7.82, p=5.55×10-4). Fatigue showed the most associations, including 3-hydroxystachydrine (β=1.02, p=3.79×10-8) and N-acetylglycine (β=0.927, p=5.89×10-5), and pain were associated with inulin (β=-3.19, p=5.39×10-5). Associations between race and PNS–metabolite interactions revealed unique patterns for Black women, particularly for sleep disturbances, pain and fatigue. Taurine/hypotaurine and cysteine metabolism were the most impacted pathways in the enrichment analysis.

**Conclusions:**

These findings highlight distinct metabolite profiles underlying PNS and suggest that sulfur amino acid- and oxidative stress-related pathways may contribute to symptom variability. Specific metabolites may reflect underlying metabolic pathway differences across racial groups. These results provide a foundation for future mechanistic studies and metabolically targeted interventions in cancer survivorship.

## Introduction

Breast cancer is the most frequently diagnosed cancer in women (1), with an estimated 4.3 million women in the United States with a history of invasive breast cancer (2). Advances in early detection and treatment have substantially improved survival; however, many survivors experience persistent pain, fatigue, anxiety, depression, and sleep disturbances, collectively known as psychoneurological symptoms (PNS), that negatively affect quality of life and work outcomes (3–5). Across the cancer treatment trajectory and into long-term survivorship (1 to 6 years post treatment), approximately 40% to 90% of women report clinically meaningful PNS burden (3, 5, 6). Psychoneurological symptoms represent a biologically embedded phenotype shaped by immune, metabolic, and sociobiological factors across the cancer trajectory. Importantly, emerging studies suggest that Black/African American women with breast cancer may experience higher levels of PNS, including anxiety, pain, and fatigue, following treatment compared with White women (7, 8). These disparities, coupled with increased mortality risk and survivorship challenges, underscore the critical need to better understand the biological processes underlying PNS among breast cancer survivors (BCS) particularly, for Black/African American women (9). Race captures the cumulative impact of structural, environmental, and biological exposures that may affect metabolic regulation and symptom vulnerability.

Although early efforts focused on chemotherapy as the causative factor in the development and persistence of PNS, more recent research has acknowledged the multi-factorial basis for PNS. Our research and that of others has identified that PNS are present prior to the receipt of chemotherapy and are associated with complex patterns of pro- and anti-inflammatory activation (10–12). Indeed, research has shown that chemotherapy results in elevated levels of circulating proinflammatory cytokines in the short term, but levels typically decline over the subsequent months (13). Although this knowledge has increased understanding of the relationship between inflammatory perturbations and PNS, the composition of biochemical mediators has not yet been clearly identified nor has the biochemical involvement of other metabolites and metabolic pathways been elucidated (14).

Metabolomics offers a systems level approach to identifying biomarkers associated with processes and symptom burden, with potential utility for early disease detection and monitoring of disease progression (15). In BCS, the tryptophan/kynurenine pathway has been implicated, involving various metabolites such as 3-hydroxykynurenine (3HK), 3-hydroxyanthranilic acid, quinolinic acid, and tryptophan (TRP) (16, 17). Due to persistent inflammatory activation resulting from breast cancer and its treatments, the enzyme indoleamine 2, 3-dioxygenase (IDO) degrades TRP, which is an essential amino acid, leading to reduced serotonin synthesis and increased production of neurotoxic metabolites (16, 17). This imbalance between the neuroprotective and neurodegenerative metabolites may contribute to behavioral symptoms such as depression, anxiety, sleep disturbances, pain, and fatigue. Previously, our group reported pilot study findings from serum metabolome analysis conducted before initial chemotherapy and one to two weeks after the final chemotherapy infusion in women with early-stage breast cancer (18). The study found that levels of pain, fatigue, and depression increased following chemotherapy, and these symptoms were correlated with higher concentrations of acetyl-L-alanine and indoxyl sulfate as well as lower levels of 5-oxo-L-proline (18). Additionally, our group further evaluated metabolite changes prior to chemotherapy initiation and one year after treatment, finding significant alterations in lysine degradation, branched-chain amino acid (BCAA) synthesis, linoleic acid metabolism, tyrosine metabolism, and the biosynthesis of unsaturated fatty acids, with BCAA synthesis and lysine degradation upregulated and unsaturated fatty acid biosynthesis and linoleic acid metabolism downregulated one year after chemotherapy (19).

Although previous studies have identified relationships between PNS and metabolomics, it is important to recognize the various factors that alter metabolite regulation in the body, such as age, diet, environment, and race, have led to inconsistencies among previous studies. Specifically, Black/African American women have a disproportionately larger cancer burden as indicated by earlier onset, higher mortality, and lower survival rates compared to other racial/ethnic groups (1, 20). A recent study reported differences in several metabolites between White and Black women, identifying 26 metabolites that differed significantly between the two groups, with Black women having higher levels of thymol sulfate, 2-naphthol sulfate, and 2-hydroxyfluorene sulfate (derivatives of polycyclic aromatic hydrocarbons) and White women having higher xanthin metabolites (21). However, limited studies have focused on racial differences in metabolomics in relation to PNS among BCS.

While previous studies have identified differences in metabolites between women with breast cancer and healthy controls as well as cross-sectional associations between metabolites and PNS, few have examined changes in PNS and their relationship to metabolites over time across the treatment trajectory. To address this gap, this study aimed to examine the longitudinal association between metabolite changes and levels of depression, anxiety, sleep disturbance, pain, and fatigue over time and explored the effect of the interaction between race and metabolites on PNS. This study focused on elucidating the variables that may contribute to PNS by integrating novel metabolomics data with our behavioral responses, such as symptom outcome measures. This new information may be used for development of targeted approaches for future clinical applications.

## Materials and methods

### Study design and sample

This secondary analysis used data from the parent study (EPIGEN), a two-year longitudinal, prospective investigation of PNS and their associations with inflammation and epigenetic changes in women diagnosed with early-stage breast cancer. In the parent study, data was collected at five timepoints: before chemotherapy (T1), at the midpoint of chemotherapy (fourth treatment, T2), and at six months (T3), one year (T4), and two years after chemotherapy initiation (T5). This study used data collected at T1, T2, T3, and T4. At each time point, PNS, including anxiety, depression, fatigue, pain, and sleep disturbances, and blood samples were obtained. The study was IRB-approved (the Virginia Commonwealth University IRB #HM 13194 CR4 and University of Florida RB201400083). Participants were recruited from a National Cancer Institute-designated comprehensive cancer center affiliated with Virginia Commonwealth University Health System and multiple regional collaborative sites. Inclusion criteria were women (a) aged 21 years or older, (b) diagnosed with early-stage breast cancer (Stage I-IIIA), and (c) scheduled to start chemotherapy treatment. Exclusion criteria included (a) a history of previous cancer or chemotherapy, (b) immune system disorders such as systemic lupus erythematosus and multiple sclerosis, and (c) neurocognitive or psychological problems (e.g., dementia, active psychosis). All participants provided written informed consent.

### Measures

Participant sociodemographic and clinical data were collected through interviews and medical records, including age, race/ethnicity, education, employment, marital status, income, body mass index (BMI), menopausal status, lifestyle factors (smoking and alcohol consumption), tumor characteristics (grade and stage), receptor status (estrogen receptor [ER], human epidermal growth factor receptor 2 [HER2]), chemotherapy regimen, and radiation therapy.

Psychoneurological symptoms, such as pain, sleep disturbance, fatigue, depression, and anxiety, were assessed at four time points using validated and reliable instruments. Pain was measured with the 9-item Brief Pain Inventory-short form (BPI-SF), which evaluates severity (subscales: worst, least, average, and right now) and interference (subscales: general activity, mood, walking ability, normal work, relations with other people, sleep, and enjoyment of life) (Cronbach’s α = 0.86 - 0.96) (22). Sleep disturbance was assessed using the 21-item General Sleep Disturbance Scale (GSDS), which evaluates difficulty falling asleep, waking up during sleep, early morning awakenings, sleep quality, sleep quantity, daytime sleepiness, and the use of substances, with scores ≥ 43 indicating significant disruptions (Cronbach’s α = 0.79) (23, 24). Fatigue was measured by the 9-item Brief Fatigue Inventory (BFI), which captures severity (right now, usual, and worst) and functional impact on daily activities (general activity, mood, walking ability, normal work, relations with other people, and enjoyment of life) (Cronbach’s α = 0.95) (25, 26). Anxiety and depression were evaluated using the 14-item Hospital Anxiety and Depression Scale (HADS), with separate subscales for each (Cronbach’s α for anxiety = 0.82, Cronbach’s α for depression = 0.82) (27, 28).

### Untargeted metabolomics procedure

Details of the metabolomic profiling process are described in our previous studies (18, 19). Blood samples (N = 74) were processed at the University of Florida Southeast Center for Integrated Metabolomics (SECIM) for protein precipitation with organic solvents. After centrifugation and solvent evaporation, residues were reconstituted in water containing injection standards for polar compound analysis. Untargeted metabolomics was performed on a Thermo Q-Exactive Orbitrap mass spectrometer coupled with Dionex ultra high-performance liquid chromatography (UHPLC), operating in positive and negative electrospray ionization modes. Each 25 µL sample was mixed with internal standard solution (5 µL) and a solvent mixture (acetonitrile:methanol:acetone with an 8:1:1 ratio, 200 µL), then vortexed, incubated for 30 minutes at 4 °C, centrifuged at 20,000 rcf for 10 minutes, dried under nitrogen, and reconstituted in injection standards. Quality control included blanks. Data processing was performed using MZmine 2.53 for alignment and MetaboAnalyst 3.0 for functional analysis, applying a 10-ppm mass tolerance, filtering noise and adducts, and retaining only significant metabolites (false discovery rate [FDR] < 0.05). Metabolite identification was performed based on the m/z value, retention time, and ionization polarity. Additional compounds were annotated *post hoc* metabolites by matching *m/z* and retention time to an in-house library. The subclass and superclass of the metabolites were identified using the Human Metabolome Database (HMDB).

### Statistical analysis

Descriptive statistics were calculated by means, standard deviations (SD), medians, and interquartile ranges (IQR) for continuous variables. Counts and percentages were computed for categorical variables. The final analysis included 74 participants with complete covariate and five baseline symptom measures, including pain severity score, fatigue severity score, total anxiety score, total depression score, and total sleep disturbance score. The covariates of interest included age, race, education level, employment status, income level, marital status, menstrual status, current smoking status, current alcohol consumption status, and BMI. These covariates were compared between White and Black/African American racial groups using Fisher’s exact test for categorical variables and the Wilcoxon rank-sum test for continuous variables.

#### Relationship between metabolites and anxiety, depression, and sleep disturbance

To reduce the impact of extreme values, natural log-transformation was applied to the metabolite levels. Generalized estimating equations (GEE) models were used to study the relationship between symptoms and metabolite levels over time and their interactions with race/ethnicity in this longitudinal dataset, which includes four time points (baseline and 3 follow-ups). Individual models were created for each of the 2,395 metabolites. The PNS were included as response variables and log-transformed metabolite levels and their interactions with race/ethnicity as explanatory variables, with adjustments for all covariates of interest. The assumption that data were missing completely at random (MCAR) was applied in the analysis. Variable selection for all models was performed using backward selection based on the Quasi-Likelihood Information Criterion (QIC).

#### Relationship between metabolites and pain and fatigue

The pain severity and fatigue severity scores in this study are zero inflated, indicating a large proportion of subjects having zero score values. Poisson and negative binomial regressions have been used commonly to model count data, but when zero-inflated Poisson data appear, these approaches will generate biased results and zero-inflated Poisson models are called for. However, the longitudinal feature of this data makes the implementation of such models originally designed for cross-sectional data even more challenging. Furthermore, the scores in this study were obtained through averaging across different questions, which are no longer count values and could not be modeled using Poisson. Therefore, a two-stage analysis was implemented for this study. This approach treated the data as a mixture of two distributions: one that generates zero and another that generates non-zero scores. In the first stage, we applied GEE with logit link to model the association of longitudinal binary data (zero vs. non-zero scores) with metabolites levels over time (baseline and 3 follow-ups) and their interactions with race/ethnicity, adjusted by all covariates of interest. In the second stage, we focused on the non-zero data and applied GEE with Gaussian link to model the association of non-zero scores with metabolites levels over time and their interactions with race/ethnicity in a similar longitudinal fashion. The same analytical pipelines were used for both stages. All 2,395 metabolites were log-transformed and individually tested as exposures in GEE models. The analysis assumed data followed MCAR assumption. The QIC was used for backward selection for all models.

To address multiple comparisons, the FDR method was used to identify significant metabolites, with an adjusted *p*-value threshold of < 0.05. All analyses were conducted using R version 4.4.1 (R Project). The metabolite set enrichment analysis was performed using the Enrichment Analysis program in MetaboAnalyst 6.0.

## Results

### Characteristics of the sample

The current study included 74 participants (White: *n* = 52; Black/African American: *n* = 22), with a mean age of 51.3 years (SD = 10.3). Most participants had completed education beyond high school (78.3%) and were non-smokers (79.7%). More than half were employed full-time (55.4%), reported an annual income of $60,000 or higher (54.0%), were married or partnered (62.2%), were postmenopausal (56.8%), and currently consumed alcohol (55.4%). On average, participants were obese (BMI = 29.9 ± 7.5 kg/m²). White participants were more likely to be older (*p* = .010), employed (*p* = .002), more educated (*p* = .014), married or partnered (*p* = .027), and to consume alcohol (*p* = .002). Additionally, participants had cancer diagnoses of stage I (27.0%), IIA (41.9%), IIB (20.3%), and IIIA (10.8%). Most participants were HER2-negative (81.8%) and estrogen receptor-positive (58.1%), had no neoadjuvant treatment (90.5%), and received radiation therapy (78.4%). Half of the participants were treated with the TAC regimen (docetaxel, doxorubicin, and cyclophosphamide; 48.6%), followed by the TC regimen (docetaxel and cyclophosphamide; 29.7%). Menstrual status, smoking, BMI, and cancer-related factors did not differ significantly between the two groups (**Table 1**).

**Table 1:** Sample demographics and cancer-related factors.

Characteristic	Overall (N = 74)	Black/African American (N = 22)	White (N = 52)	*P*-value^1^
Age (years), mean (SD)	51.3 (10.3), 23-71	46.6 (8.9)	53.3 (10.2)	**0.010**
Ethnicity				0.21
Hispanic or Latino	3 (4.1%)	2 (9.1%)	1 (1.9%)	
Not Hispanic or Latino	71 (95.9%)	20 (90.9%)	51 (98.1%)	
Education				**0.014**
Didn’t finish High School	7 (9.5%)	5 (22.7%)	2 (3.9%)	
High School Diploma	9 (12.2%)	4 (18.2%)	5 (9.6%)	
Any education beyond High School	58 (78.3%)	13 (59.1%)	45 (86.5%)	
Employment				**0.002**
Full-time	41 (55.4%)	9 (40.9%)	32 (61.5%)	
Part-time	5 (6.8%)	2 (9.1%)	3 (5.8%)	
Retired	10 (13.5%)	0 (0.0%)	10 (19.2%)	
Student	1 (1.4%)	1 (4.5%)	0 (0.0%)	
Unemployed	17 (23.0%)	10 (45.5%)	7 (13.5%)	
Income				**< 0.001**
< $15,000	11 (14.9%)	9 (40.9%)	2 (3.8%)	
$15,000 ~ $29,999	8 (10.8%)	3 (13.6%)	5 (9.6%)	
$30,000 ~ $44,999	8 (10.8%)	5 (22.7%)	3 (5.8%)	
$45,000 ~ $59,999	7 (9.5%)	3 (13.6%)	4 (7.7%)	
$60,000 ~ $74,999	7 (9.5%)	1 (4.5%)	6 (11.5%)	
$75,000 ~ $89,999	11 (14.9%)	0 (0.0%)	11 (21.2%)	
$90,000 ~ $104,999	8 (10.8%)	0 (0.0%)	8 (15.4%)	
≥ $105,000	14 (18.8%)	1 (4.5%)	13 (25.0%)	
Marital Status				**0.027**
Divorced/Separated	18 (24.3%)	7 (31.8%)	11 (21.2%)	
Married/Partner	46 (62.2%)	9 (40.9%)	37 (71.2%)	
Single/Never married	10 (13.5%)	6 (27.3%)	4 (7.7%)	
Menstrual Status				0.39
Peri-menopausal	6 (8.1%)	2 (9.1%)	4 (7.7%)	
Post-menopausal	42 (56.8%)	10 (45.5%)	32 (61.5%)	
Premenopausal	26 (35.1%)	10 (45.5%)	16 (30.8%)	
Currently Smoking				0.12
Yes	15 (20.3%)	7 (31.8%)	8 (15.4%)	
No	59 (79.7%)	15 (68.2%)	44 (84.6%)	
Currently Drinking				**0.002**
Yes	41 (56.2%)	6 (28.6%)	35 (67.3%)	
No	32 (43.8%)	15 (71.4%)	17 (32.7%)	
BMI (kg/m^2^), mean (SD)	29.9 (7.5), 19.1-54.3	31.5 (7.4)	29.2 (7.6)	0.12
Grade				0.15
1	5 (6.8%)	1 (4.5%)	4 (7.7%)	
2	28 (37.8%)	12 (54.5%)	16 (30.8%)	
3	41 (55.4%)	9 (40.9%)	32 (61.5%)	
Stage				0.18
I	20 (27.0%)	5 (22.7%)	15 (28.8%)	
IIA	31 (41.9%)	11 (50.0%)	20 (38.5%)	
IIB	15 (20.3%)	6 (27.3%)	9 (17.3%)	
IIIA	8 (10.8%)	0 (0.0%)	8 (15.4%)	
Estrogen receptor positive				0.20
Yes	43 (58.1%)	10 (45.5%)	33 (63.5%)	
No	31 (41.9%)	12 (54.5%)	19 (36.5%)	
HER-2 positive				0.33
Yes	14 (18.9%)	6 (27.3%)	8 (15.4%)	
No	60 (81.1%)	16 (72.7%)	44 (84.6%)	
Neoadjuvant				0.19
Yes	7 (9.6%)	4 (18.2%)	3 (5.9%)	
No	66 (90.4%)	18 (81.8%)	48 (94.1%)	
Chemotherapy regimen				0.38
AC	2 (2.7%)	0 (0.0%)	2 (3.8%)	
CMF	2 (2.7%)	0 (0.0%)	2 (3.8%)	
TAC	36 (48.6%)	10 (45.5%)	26 (50.0%)	
TC	22 (29.7%)	6 (27.3%)	16 (30.8%)	
TCH	12 (16.2%)	6 (27.3%)	6 (11.5%)	
Radiation				0.36
Yes	58 (78.4%)	19 (86.4%)	39 (75.0%)	
No	16 (21.6%)	3 (13.6%)	13 (25.0%)	

^1^Wilcoxon rank sum test and Fisher’s exact test were used.

BMI, body mass index; HER2, human epidermal growth factor receptor 2.

Chemotherapy regimen AC, doxorubicin and cyclophosphamide; CMF, cyclophosphamide, methotrexate, and 5-fluorouracil; TAC, docetaxel, doxorubicin, and cyclophosphamide; TC, docetaxel and cyclophosphamide; TCH, docetaxel, carboplatin, and trastuzumab.

Bold p-values indicate p < .05.

### Psychoneurological symptom characteristics

#### Depression, anxiety, and sleep disturbance

**Figure 1:**
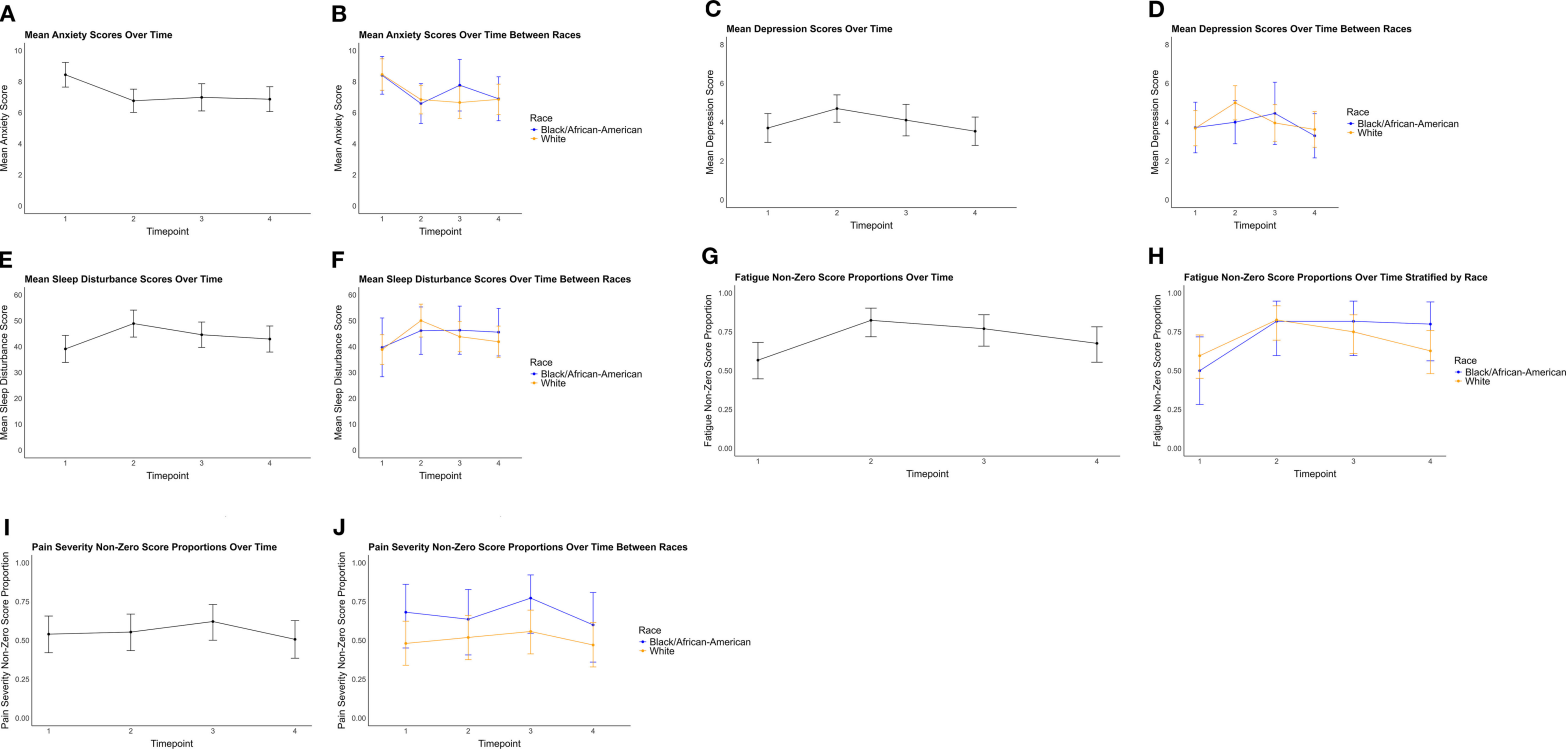
Psychoneurological symptom profiles in breast cancer survivors over one year. **(A)** Mean anxiety score measured by the Hospital Anxiety and Depression Scale (HADS) over time; **(B)** Mean anxiety score measured by the HADS over time, comparing White and Black participants (blue = Black breast cancer survivors, orange = White breast cancer survivors); **(C)** Mean depression score measured by the HADS over time; **(D)** Mean depression score measured by the HADS over time between races; **(E)** Mean sleep disturbance score measured by the General Sleep Disturbance Scale (GSDS) over time; **(F)** Mean sleep disturbance score measured by the GSDS over time between races; **(G)** Mean proportion of non-zero fatigue scores measured by the Brief Fatigue Inventory (BFI) over time; **(H)** Mean proportion of non-zero fatigue scores measured by the BFI over time between races; **(I)** Mean proportion of non-zero pain scores measured by the Brief Pain Inventory (BPI) over time; **(J)** Mean proportion of non-zero pain scores measured by the BPI over time between races. (T1 = baseline, T2 = mid-point of chemotherapy, T3 = 6-month follow-up, T4 = 1-year follow-up).

As seen in **Figure 1**, the mean anxiety score was the highest at baseline (8.4 ± 0.8) and then decreased to 6.8 ± 0.7 at mid-point of chemotherapy. Scores remained stable from the mid-point of chemotherapy to 6 months, with a gradual decline until 1-year follow-up. The trajectories of anxiety in White BCS and Black BCS indicated that differences were small and comparable reductions over time. The mean sleep disturbance score measured by GSDS showed an initial increase from baseline (39.1 ± 5.2) to the mid-point of chemotherapy, peaking at 48.9 ± 5.2, followed by a gradual decline through 6 months and one year after initiating chemotherapy. Similar baseline sleep disturbances for White and Black BCS were observed, and at mid-point chemotherapy White BCS experienced a pronounced increase in sleep disturbance (50.1 ± 6.3) while Black BCS showed a smaller rise (46.2 ± 9.2). Scores for White BCS declined, and both groups showed similar levels of sleep disturbance. The mean depression score was 3.7 ± 0.7, increasing to its peak of 4.7 ± 0.7 at the mid-point chemotherapy. After this peak, scores declined steadily over timepoints. White and Black BCS showed similar mean depression scores around 3.0 at baseline, but scores for White BCS increased at the mid-point chemotherapy, peaking at 5.0 ± 0.9. Both groups demonstrated a slight decline, though the White BCS remained higher than Black BCS. By one year and two years after initiating chemotherapy, mean depression scores decreased for both groups toward similar levels near 2.5-3.0.

#### Pain and fatigue

Forty out of 74 participants (54.1%; 95% CI: 42.1% - 65.7%) reported non-zero pain severity scores at baseline and remained relatively stable at the mid-point of chemotherapy (34 participants reported zero scores at baseline). A slight increase was observed at 6 months after initiating chemotherapy, followed by a decline at one year to 50.7%. The proportion of Black BCS reporting non-zero pain was higher than that of White BCS at baseline, and this pattern persisted across all timepoints, with Black BCS consistently showing greater pain prevalence. White BCS exhibited relatively stable proportions, ranging between 48.1% and 55.8%. The proportion of participants reporting non-zero fatigue was 56.8% at baseline, increasing to 82.4% at the mid-point of chemotherapy. After this peak, the proportion declined gradually to 77.0% (57/74; 95% CI: 65.8%-86.0%) at 6 months and 67.6% (48/71; 95% CI: 55.5%-78.2%) at one year. Thirty-one out of 52 White BCS (59.6%; 95% CI: 45.1%-73.0%) reported fatigue compared with 11 out of 22 Black BCS (50.0%; 95% CI: 28.2%-71.8%) at baseline. Both groups increased at the mid-point of chemotherapy, converging near 82%. Black BCS remained relatively stable, with proportions between 80% and 82% through the 1-year follow-up, whereas White BCS exhibited a decline after the mid-point, dropping to 62.7% (39/52; 95% CI: 48.1%-75.9%) at the 1-year follow-up (**Figure 1**).

Among participants who reported non-zero pain, the mean scores were 3.0 ± 0.7 at baseline (Black: 3.8 ± 1.1; White: 2.5 ± 0.8), 3.3 ± 0.6 at mid-point chemotherapy (Black: 4.4 ± 0.9; White: 2.7 ± 0.7), 3.6 ± 0.6 at the 6-month follow-up (Black: 4.5 ± 1.1; White: 3.1 ± 0.6), and 3.3 ± 0.8 at the 1-year follow-up (Black: 4.7 ± 1.7; White: 2.7 ± 0.8). Among participants who reported non-zero fatigue, the mean scores were 3.5 ± 0.8 at baseline (Black: 4.8 ± 1.7; White: 3.0 ± 0.6 0.8), 4.0 ± 0.6 at mid-point chemotherapy (Black: 4.1 ± 1.3; White: 4.0 ± 0.7), 3.7 ± 0.7 at the 6-month follow-up (Black: 4.4 ± 1.3; White: 3.4 ± 0.8), and 3.4 ± 0.6 at the 1-year follow-up (Black: 3.5 ± 1.3; White: 3.3 ± 0.7). Overall, participants who reported non-zero pain and fatigue experienced mild levels of both, with Black BCS showing higher pain and fatigue compared to White BCS.

### Associations between metabolites and psychoneurological symptoms

We conducted the GEE to obtain an understanding of the longitudinal association between metabolites and PNS over time in BCS. In the model adjusted for demographic and lifestyle-related variables, there were 140 metabolites significantly associated with anxiety and sleep disturbance (**Supplementary Table 1**) out of 2,395 metabolites, among which 38 metabolites were identified by name. Decreased anxiety was significantly associated with 2-aceto-2-hydroxy-butanoate (β = -2.40, *p* = 5.79×10^-6^, *q* = 6.87×10^-3^), 1-pyrrolidinecarboxaldehyde (β = -0.753, *p* = 8.61×10^-6^, *q* = 6.87×10^-3^), and 2-Methyl-3-(propyldithio)furan (β = -1.46, *p* = 1.57×10^-4^, *q* = 2.68×10^-2^), while increased anxiety was significantly associated with 2,4,6-triethyl-1,3,5-oxadithiane (β = 1.15, *p* = 1.08×10^-4^, *q* = 2.32×10^-2^). Increased sleep disturbance was significantly associated with 4,6-O-ethylidene-D-glucose (β = 9.83, *p* = 4.82×10^-6^, *q* = 2.88×10^-3^), octyl gallate (β = 6.40, *p* = 1.76×10^-4^, *q* = 3.74×10^-2^), pyridine N-oxide (β = 12.8, *p* = 4.11×10^^-^4^, *q* = 4.10×10^-2^), and 5-hydroxytryptophol (β = 7.82, *p* = 5.55×10^-4^*, q* = 4.57×10^-2^), while decreased sleep disturbance was significantly associated with 1-pyrrolidinecarboxaldehyde (β = -4.26, *p* = 1.27 × 10^-4^, *q* = 3.39×10^-2^). There were no identified metabolites associated with depression (**Table 2**).

**Table 2:** Associations between metabolites and psychoneurological symptoms.

Symptom	Metabolite	Sub Class	Super Class	Coefficient	P-value	FDR value
Anxiety	2-aceto-2-hydroxy-butanoate	Short-chain keto acids and derivatives	Organic acids and derivatives	-2.40	5.79 × 10^-6^	6.87 × 10^-3^
	1-Pyrrolidinecarboxaldehyde	N/A	Organoheterocyclic compounds	-0.753	8.61 × 10^-6^	6.87 × 10^-3^
	2,4,6-Triethyl-1,3,5-oxadithiane	Monothioacetals	Organosulfur compounds	1.15	1.08 × 10^-4^	2.32 × 10^-2^
	2-Methyl-3-(propyldithio)furan	N/A	Organoheterocyclic compounds	-1.46	1.57 × 10^-4^	2.68 × 10^-2^
	2,4,6-Triethyl-1,3,5-oxadithiane	Monothioacetals	Organosulfur compounds	1.13	1.70 × 10^-4^	2.71 × 10^-2^
Sleep disturbances	4,6-O-Ethylidene-D-glucose	N/A	Organoheterocyclic compounds	9.83	4.82 × 10^-6^	2.88 × 10^-3^
	1-Pyrrolidinecarboxaldehyde	N/A	Organoheterocyclic compounds	-4.26	1.27 × 10^-4^	3.39 × 10^-2^
	Octyl gallate	Benzoic acids and derivatives	Benzenoids	6.40	1.76 × 10^-4^	3.74 × 10^-2^
	Pyridine N-oxide	Pyridinium derivatives	Organoheterocyclic compounds	12.8	4.11 × 10^-4^	4.10 × 10^-2^
	5-Hydroxytryptophol	Hydroxyindoles	Organoheterocyclic compounds	7.82	5.55 × 10^-4^	4.57 × 10^-2^
Fatigue	3-Hydroxystachydrine	Amino acids, peptides, and analogues	Organic acids and derivatives	1.02	3.79 × 10^-8^	2.27 × 10^-5^
	4-hydroxystachydrine	Amino acids, peptides, and analogues	Organic acids and derivatives	0.985	8.10 × 10^-8^	3.53 × 10^-5^
	2-(2-hydroxybenzoyl)hydrazinecarboxamide	N/A	N/A	1.09	1.18 × 10^-7^	4.02 × 10^-5^
	Cys-Ser	Amino acids, peptides, and analogues	Organic acids and derivatives	-1.00	1.48 × 10^-6^	3.22 × 10^-4^
	Ethylparaben sulfate	Arysulfates	Organic acids and derivatives	-0.773	2.99 ×10^-6^	5.12 × 10^-4^
	NA-Arg 18:3	Amino acids, peptides, and analogues	Organic acids and derivatives	-0.492	1.84 × 10^-5^	1.91 × 10^-3^
	Hippurate	Benzoic acids and derivatives	Benzenoids	-0.944	2.46 × 10^-5^	2.26 × 10^-3^
	2-hydroxybenzenesulfonic acid	Benzenesulfonic acids and derivatives	Benzenoids	-0.843	2.86 × 10^-5^	2.54 × 10^-3^
	O-acetylserine; lactoylglycine	Amino acids, peptides, and analogues	Organic acids and derivatives	0.932	4.66 × 10^-5^	3.60 × 10^-3^
	N-acetylglycerine	Amino acids, peptides, and analogues	Organic acids and derivatives	0.927	5.89 × 10^-5^	4.41 × 10^-3^
	N-acetylvaline; valerylglycine	Amino acids, peptides, and analogues	Organic acids and derivatives	0.610	6.75 × 10^-5^	4.75 × 10^-3^
	FAHFA 18:1(9Z)/3O-9:0	Fatty acids and conjugates	Lipids and lipid-like molecules	-1.54	3.08 × 10^-4^	1.83 × 10^-2^
	3-sulfino-L-alanine	Amino acids, peptides, and analogues	Organic acids and derivatives	0.958	3.72 × 10^-4^	2.07 × 10^-2^
	Lactoylvaline	Amino acids, peptides, and analogues	Organic acids and derivatives	0.608	4.26 × 10^-4^	2.32 × 10^-2^
	3-Methyldioxyindole	Indolines	Organoheterocyclic compounds	-0.902	5.14 × 10^-4^	2.67 × 10^-2^
	Methylhippurate, formylphenylalanine	Amino acids, peptides, and analogues	Organic acids and derivatives	0.963	7.74 × 10^-4^	3.28 × 10^-2^
	cis-2-Methylaconitate	Tricarboxylic acids and derivatives	Organic acids and derivatives	0.546	7.76 × 10^-4^	3.28 × 10^-2^
	Methylhippurate, formylphenylalanine	Amino acids, peptides, and analogues	Organic acids and derivatives	0.725	7.89 × 10^-4^	3.28 × 10^-2^
	Homovanillic acid sulfate	Arylsulfates	Organic acids and derivatives	0.474	8.51 × 10^-4^	3.45 × 10^-2^
	Deoxyribose 1-phosphate	Carbohydrates and carbohydrate conjugates	Organic oxygen compounds	1.18	1.03 × 10^-3^	3.73 × 10^-2^
	Malondialdehyde	Carbonyl compounds	Organic oxygen compounds	1.10	1.10 × 10^-3^	3.82 × 10^-2^
	Methylguanidine	Guanidines	Organic nitrogen compounds	1.38	1.17 × 10^-3^	3.95 × 10^-2^
	N-acetylneuraminol	N/A	N/A	1.10	1.29 × 10^-3^	4.11 × 10^-2^
	D-erythro-L-galacto-Nonulose	Carbohydrates and carbohydrate conjugates	Organic oxygen compounds	0.809	1.38 × 10^-3^	4.25 × 10^-2^
	3-hexenedioic acid	Fatty acids and conjugates	Lipids and lipid-like molecules	1.05	1.53 × 10^-3^	4.65 × 10^-2^
	2-deoxyribonic acid	Beta hydroxy acids and derivatives	Organic acids and derivatives	1.08	1.66 × 10^-3^	4.91 × 10^-2^
Pain	Inulin	N/A	Organic polymers	-0.319	5.39 × 10^-5^	3.23 × 10^-2^
	NA-Arg 18:3	Amino acids, peptides, and analogues	Organic acids and derivatives	-0.502	9.73 × 10^-5^	4.66 × 10^-2^

Fatigue exhibited the largest number of significant associations. 3-hydroxystachydrine (β = 1.02, *p* = 3.79×10^-8^, *q* = 2.27×10^-5^), 4-hydroxystachydrine (β = 0.985, *p* = 8.10×10^-8^, *q* = 3.53×10^-5^), and 2-(2-hydroxybenzoyl)hydrazinecarboxamide (β = 1.09, *p* = 1.18×10^-7^, *q* = 4.02×10^-5^) were significantly associated with increased fatigue. Additionally, positive associations with fatigue were observed for amino acid derivatives, such as N-acetylglycine (β = 0.927, *p* = 5.89×10^-5^, *q* = 4.41×10^-3^) and O-acetylserine;lactoylglycine (β = 0.932, *p* = 4.66×10^-5^, *q* = 3.60×10^-3^). Ethylparaben sulfate (β = −0.773, *p* = 2.99×10^-6^, *q* = 5.12×10^-4^) and Hippurate (β = −0.944, *p* = 2.46×10^-5^, *q* = 2.26×10^-3^) showed significant associations with decreased fatigue. Significant associations with decreased pain were found for Inulin (β = −3.19, *p* = 5.39×10^-5^, *q* = 3.23×10^-2^) and NA-Arg 18:3 (β = −0.502, *p* = 9.73×10^-5^, *q* = 4.66×10^-2^). These results for fatigue and pain were derived from the second stage analysis focusing on participants with non-zero fatigue and pain scores. No significant associations were observed between the odds of non-zero fatigue and pain scores and any metabolites.

### Associations between race and psychoneurological symptom-metabolite interactions

As shown in **Table 3**, the adjusted GEE model revealed an association between race and PNS-metabolite interactions. Of the 32 metabolites listed in **Supplementary Table 2**, 12 were identified by name. For sleep disturbances, Black BCS participants showed a significantly higher association with 1-methylcytosine (β = 11.8, *p* = 5.86 × 10^-8^, *q* = 1.40×10^-4^), but a weaker association with 6-hydroxyoctanoylcarnitine (β = –8.19, *p* = 3.92 × 10^-5^, *q* = 2.49×10^-2^) compared to White participants. For pain, eight metabolites demonstrated significant associations. N-lactoyl tryptophan (β = –2.28, *p* = 2.20 × 10^-5^, *q* = 2.47×10^-2^), nicotyrine (β = –1.69, *p* = 1.76 × 10^-4^, *q* = 3.91×10^-2^), 2-dimethylaminoethylphosphonic acid (β = –0.986, *p* = 2.80 × 10^-4^, *q* = 4.08×10^-2^), 3-oxohexacosanoic acid (β = –1.45, *p* = 2.90 × 10^-4^, *q* = 4.08×10^-2^), and 2-methoxy-3,5-dimethylpyrimidine (β = –1.86, *p* = 3.59 × 10^-4^, *q* = 4.77×10^-2^), were less strongly associated with Black participants compared to White women. In contrast, 5-hydroxyindoleacetylglycine (β = 9.82, *p* = 3.10 × 10^-7^, *q* = 2.48×10^-4^), triuret in negative ionization mode (β = 0.651, *p* = 2.69 × 10^-4^, *q* = 4.08×10^-4^), and triuret in positive ionization mode (β = 0.746, *p* = 2.88 × 10^-4^, *q* = 4.08×10^-4^) showed stronger associations with Black participants. For fatigue, Black participants had a higher association with 5-hydroxyindoleacetylglycine (β = 9.82, *p* = 3.10×10^-7^, *q* = 2.48×10^-4^) and NA-Arg 18:3 (β = 3.94, *p* = 1.03 × 10^-4^, *q* = 4.11×10^-2^). 5-hydroxyindoleacetylglycine appeared a common metabolite across pain and fatigue. Likewise, for pain and fatigue, all results were derived from the second stage analysis focusing on the non-zero symptom scores. No significant associations were observed between the odds of non-zero scores and the interaction of race and any metabolites.

**Table 3:** Associations between psychoneurological symptoms and race-metabolite interactions.

Symptom	Metabolite	Sub class	Super class	Coefficient	*P*-value	FDR value
Sleep disturbances	1-methylcytosine	Pyrimidines and pyrimidine derivatives	Organoheterocyclic compounds	11.8	5.86×10^-8^	1.40 × 10^-4^
	6-hydroxyoctanoylcarnitine	Fatty acid esters	Lipids and lipid-like molecules	-8.19	3.92 × 10^-5^	2.49 × 10^-2^
Pain	N-lactoyl tryptophan	Amino acids, peptides, and analogues	Organic acids and derivatives	-2.28	2.20 × 10^-5^	2.47 × 10^-2^
	5-hydroxyindoleacetylglycine	Amino acids, peptides, and analogues	Organic acids and derivatives	7.73	6.91 × 10^-5^	2.47 × 10^-2^
	Nicotyrine	N/A	Organoheterocyclic compounds	-1.69	1.76 × 10^-4^	3.91 × 10^-2^
	Triuret (Negative)	N/A	N/A	0.651	2.69 × 10^-4^	4.08 × 10^-2^
	2-dimethylaminoethylphosphonic acid	N/A	N/A	-0.986	2.80 × 10^-4^	4.08 × 10^-2^
	Triuret (Positive)	N/A	N/A	0.746	2.88 × 10^-4^	4.08 × 10^-2^
	3-oxohexacosanoic acid	N/A	N/A	-1.45	2.90 × 10^-4^	4.08 × 10^-2^
	2-methoxy-3,5-dimethylpyrimidine	Pyrazines	Organohterocyclic compounds	-1.86	3.59 × 10^-4^	4.77 × 10^-2^
Fatigue	5-hydroxyindoleacetylglycine	Amino acids, peptides, and analogues	Organic acids and derivatives	9.82	3.10 × 10^-7^	2.48 × 10^-4^
	NA-Arg 18:3	Amino acids, peptides, and analogues	Organic acids and derivatives	3.94	1.03 × 10^-4^	4.11 × 10^-2^

FDR, false discovery rate. The reference is the White race.

### Metabolite set enrichment analysis

**Figure 2:**
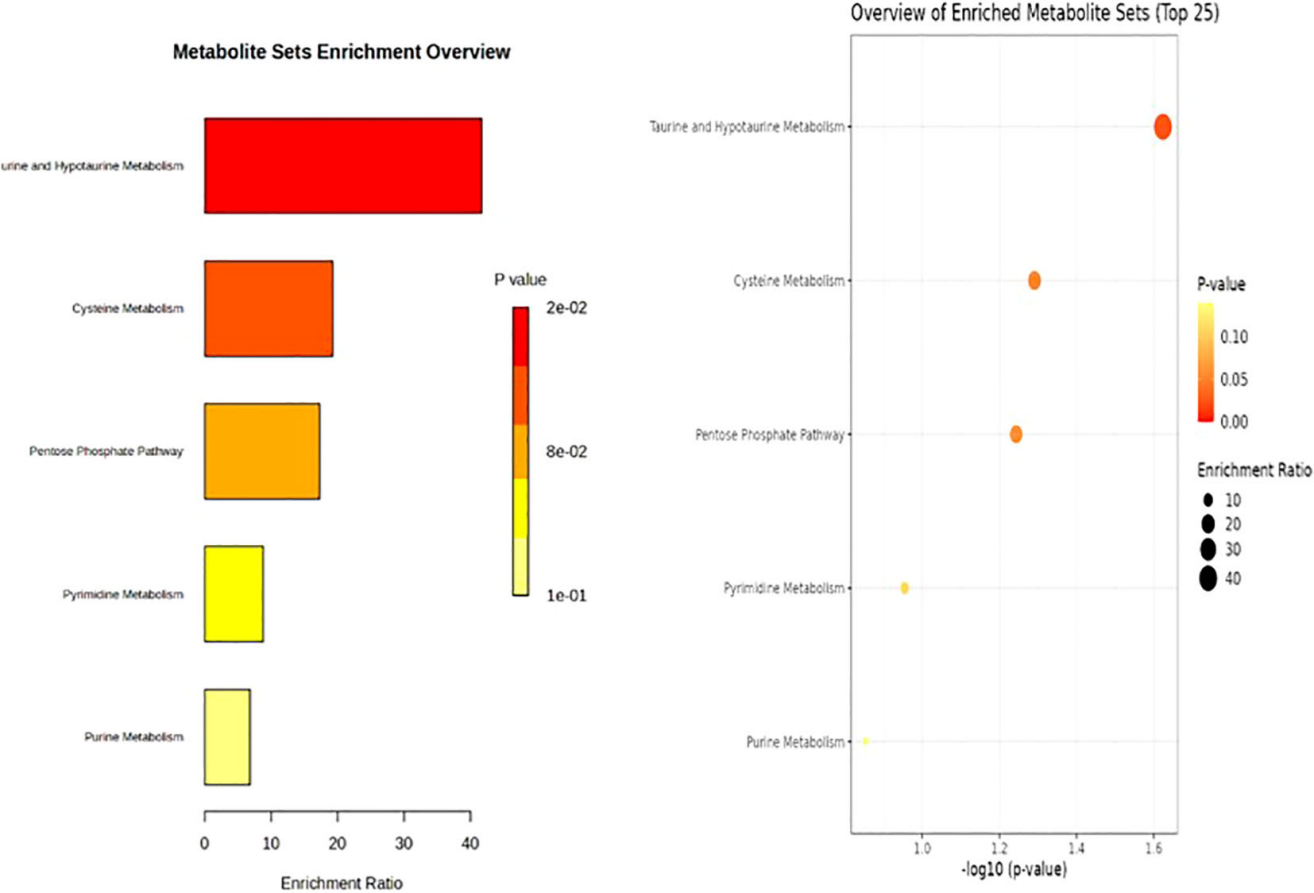
Metabolite set enrichment analysis.

**Figure 2** presents an overview of metabolite set enrichment analysis. The most significantly enriched pathway was taurine/hypotaurine metabolism, which showed the highest enrichment ratio (*p* = .027), followed by cysteine metabolism (*p* = .070) and the pentose phosphate pathway (*p* = .078). Pyrimidine metabolism (*p* = .152) and purine metabolism (*p* = .176) showed lower enrichment ratios although they were not statistically significant. These results indicate that sulfur-containing amino acid metabolism (taurine and cysteine pathways) and carbohydrate metabolism (pentose phosphate pathway) were among the most impacted metabolic processes.

## Discussion

To the best of our knowledge, this is the first study to longitudinally assess metabolomic profiles associated with PNS among breast cancer survivors using untargeted metabolomic analysis. We noted that metabolite alterations may account for symptom persistence and variability in breast cancer over time. In this study, we identified 140 metabolites significantly associated with PNS, with 38 named metabolites. Anxiety was associated with 2-aceto-2-hydroxy-butanoate and 1-pyrrolidinecarboxaldehyde, while sleep disturbance associated with 4,6-O-ethylidene-D-glucose and 5-hydroxytryptophol. Fatigue showed the most associations, including 3-hydroxystachydrine and N-acetylglycine, and pain was associated with inulin. Associations between race and PNS–metabolite interactions revealed unique patterns for Black women, notably for sleep disturbance, pain, and fatigue. Pathway enrichment highlighted taurine/hypotaurine and cysteine metabolisms.

This study showed the associations between anxiety and metabolites, such as 2-aceto-2-hydroxy-butanoate and 1-pyrrolidinecarboxaldehyde, which may implicate potential involvement of branched-chain amino acids (BCAA; valine, leucine, and isoleucine) metabolism and proline/pyrroline-5-carboxylate (P5C) redox cycling. 2-aceto-2-hydroxy-butanoate is an intermediate in the biosynthesis of BCAA, which regulate glutamate and gamma-aminobutyric acid (GABA) synthesis and effect on neurotransmission by modulating neurotransmitter synthesis such as dopamine, serotonin, and norepinephrine (29). Disruption of BCAA metabolism has been shown to change anxiety-like behaviors and stress responses in preclinical models (30, 31). Similarly, 1-pyrrolidinecarboxaldehyde is a derivative related to proline/P5C metabolism, a pathway involved in mitochondrial redox balance and glutamate cycling (32). Previous studies reported that dysregulation of proline-P5C interconversion affects emotional behaviors like anxiety, oxidative stress, and GABAergic function (33, 34).

Our study also found that increased sleep disturbance was associated with 5-hydroxytryptophol. The presence of 5-hydroxytryptophol, a metabolite of serotonin (5-hydroxytryptamine; 5-HT), is linked to altered tryptophan/serotonin metabolism with sleep disruption (35). It is formed when serotonin is deaminated by monoamine oxidase to an aldehyde intermediate, which is then reduced to 5-hydroxytryptophol (35). Because serotonin is a key precursor to melatonin, the hormone that regulates circadian rhythm and sleep-wake cycles, alteration in serotonin metabolism may impact melatonin synthesis and disturb sleep regulation (36). Davies et al. have identified that increased levels of serotonin, tryptophan, taurine, and glycerophospholipids were significantly associated with sleep deprivation, which may explain the antidepressive effect of acute sleep disorders (37).

Fatigue exhibited the most extensive set of circulating metabolite associations in this study. Especially Cys-Ser, O-acetylserine, lactoylglycine, N-acetylvaline; valerylglycine, 3-sulfino-L-alanine, lactoylvaline, methylguanidine, and NA-Arg 18:3 are linked to energy and amino acid metabolism. Aligned with our finding, in a large community cohort of 2,055 individuals, circulating metabolites associated with amino acid, lipid, and energy pathways were analyzed in relation to chronic fatigue syndromes, in which this study found that fatigue is often associated with alterations in amino acid metabolism, including changes in compounds like glutamate and N-acetylated amino acids, suggesting metabolic dysregulation underlying fatigue phenotypes (38). Similarly, Kimble et al. have identified significant correlations between plasma metabolites and fatigue scores, particularly among intermediates involved in energy metabolism pathways (e.g., tricarboxylic acid [TCA] cycle metabolites of alpha-ketoglutarate (AKG) and succinate) in chronic conditions characterized by fatigue, such as systemic lupus erythematosus (39). Filler et al. have demonstrated that disruptions in oxidative pathways can contribute to mitochondrial dysfunction and impaired energy regulation in cancer-related fatigue (40). Additionally, Hippurate, a well-established gut microbiome-derived metabolite, was associated with decreased fatigue, suggesting these metabolites may contribute to potential protective or resilience-related metabolic processes. Hippurate has been associated with favorable metabolic phenotypes, healthier dietary patterns, and reduced systemic inflammation (41).

The observed inverse association between pain severity and inulin may provide insights into the metabolic processes contributing to pain reduction among BCS. Inulin, a well-characterized fermentable dietary fiber, was strongly associated with decreased pain severity, suggesting a potential protective or immunomodulatory role of gut microbiome-related pathways (42). Prior research has demonstrated that inulin promotes the growth of beneficial microbial taxa and increases production of short-chain fatty acids (SCFAs) (43, 44), which have established anti-inflammatory and analgesic effects (45, 46). SCFAs such as butyrate have been shown to modulate nociceptive processing, reduce neuroinflammation, and regulate immune signaling, mechanisms that may help explain the association observed in our study (47).

Our findings highlight the importance of considering racial differences in metabolomic signatures associated with PNS. For sleep disturbance, Black women had a higher association with 1-methylcytosine, suggesting epigenetic processes may differentially contribute to sleep outcomes by race. DNA methylation changes have been linked to sleep disruption and shift work-related sleep phenotypes, supporting that a methylated cytosine derivative could mark sleep-related epigenetic alterations that vary by social or environmental exposures (48). For pain, Black BCS consistently showed a higher prevalence and had a weaker association with N-lactoyl-tryptophan compared to White BCS. These findings align with the previous studies. Li et al. reported that lower tryptophan levels and an elevated kynurenine-to-tryptophan ratio were significantly associated with a higher likelihood of belonging to the high-symptom subgroup, including pain, after controlling for BMI and antidepressant use, among BCS (17). In a study of 506 healthy women, 26 metabolites exhibited significant racial differences, particularly in tryptophan metabolites, which were lower in Black women than in White women (21). These findings support the hypothesis that specific metabolites might show differential associations by race, potentially reflecting underlying metabolic pathway differences that could modulate symptom phenotypes. Black women experience disproportionate breast cancer mortality and higher prevalence of aggressive subtypes, such as triple-negative breast cancer (1, 49), which may exacerbate metabolic and inflammatory dysregulation. Furthermore, social determinants of health may contribute to race-metabolite interactions. Previous research has shown that Black individuals experience higher levels of perceived stress, encounter discrimination more frequently, and are more likely to live in neighborhoods with poorer physical conditions and lower social cohesion compared with White individuals (50), which would be correlated with metabolic profiles largely influenced by diet and lifestyle (51). Butler et al. reported that more than 40% of individual metabolites or biochemical subclasses differed in abundance between Black and White participants, with most metabolites occurring at lower levels among Black individuals (51).

Sulfur-containing amino acid pathways, particularly taurine/hypotaurine metabolism, were the most significantly enriched. Taurine is known to regulate oxidative stress, calcium homeostasis, mitochondrial stability, and inflammation, and its depletion has been associated with cellular vulnerability under metabolic stress (52). Prior studies have demonstrated that taurine supplementation mitigates oxidative damage, improves mitochondrial function, and attenuates fatigue-related phenotypes in preclinical models (53–55), suggesting that impaired oxidative balance contributes to PNS. Cysteine metabolism, the second-strongest enrichment signal, further reinforces the importance of sulfur-amino-acid pathways. Cysteine is an essential precursor for glutathione synthesis, the major intracellular antioxidant system, and perturbations in the cysteine-glutathione metabolic pathway can lead to elevated oxidative stress and activation of inflammatory processes (56, 57). In cancer survivors, disrupted glutathione cycling and impaired detoxification pathways have been linked to increased fatigue, neurocognitive changes, and heightened chemotherapy-related symptom burden (58, 59). Thus, enrichment of cysteine metabolism may reflect increased metabolic demand for antioxidant defense or a compensatory response to chronic oxidative stress associated with persistent PNS. The pentose phosphate pathway (PPP), the enriched carbohydrate-related pathway, plays a central role in generating nicotinamide adenine dinucleotide phosphate (NADPH) for antioxidant regeneration and biosynthesis (60). Its enrichment is consistent with evidence that PPP activation occurs under oxidative and metabolic stress to maintain redox homeostasis (60). Upregulation of the PPP has been observed in cancer cells and in immune cells responding to inflammation (61, 62), indicating that elevated PPP activity may reflect systemic oxidative burden or chronic immune activation mechanisms frequently implicated in fatigue, sleep disturbances, and pain among cancer survivors.

### Limitations

Although this study has strengths, such as using longitudinal metabolomics and symptom behavior data, it has several limitations. First, the small sample size (N = 74) may limit statistical power, given the high dimensionality of metabolomics data. Although multiple testing corrections (FDR) were applied, the possibility of false positives remains, and findings should be interpreted as exploratory and hypothesis-generating, particularly with respect to race-metabolite interactions. Larger studies are necessary to validate these associations to improve generalizability. Second, while race was included as an interaction term, other sociodemographic and clinical factors, such as treatment regimen, comorbidities, and lifestyle behaviors (diet and physical activity), were not fully accounted for. Socioeconomic status, in particular, has been shown to be significantly associated with metabolomic variations (63). Thus, the metabolites that appeared to interact with race may have their levels influenced by socioeconomic status and/or lifestyle factors that differ between White and Black participants. For example, alcohol consumption, which was higher among the White participants in this study, is known to be associated with metabolite changes (64, 65) although no studies to date have examined the joint association of alcohol consumption, race, and metabolite changes. Race was stratified in the analysis, thereby controlling for some demographic variables, but performing more comprehensive stratified analyses will be necessary to better disentangle these interactions. Third, of the 140 metabolites significantly associated with PNS, only 38 were identified by name, and only a few could be interpreted. Many metabolites have not been extensively studied in cancer-related symptom research. Functional validation studies, including targeted metabolomics and mechanistic experiments, may help confirm their roles in symptom pathophysiology. Fourth, because this study is observational in nature, causal relationships between tumor burden, metabolite alterations, and PNS cannot be determined. Given the available data, we were unable to distinguish whether tumor burden precedes metabolite changes that, in turn, affect PNS. Therefore, our findings were interpreted as associations rather than a causal mechanism. However, preclinical studies support the possibility of such biological pathways. In mouse models, tumor burden has been shown to alter metabolomic levels, such as lactate, taurine, choline, and sugar moieties (66) perhaps through metabolic reprogramming, inflammation, energy utilization, or physiological stress (67). In human subject research, tumor burden has also been associated with reduced levels of several amino acids, lysophophatidylcholines, and diglycerides (68). In most human studies, the involvement of cancer treatment makes it difficult to distinguish the direct effects of tumor burden on metabolite changes from treatment-related metabolite changes. Lastly, pain and fatigue were analyzed differently from the other symptoms using a two-stage approach because many participants reported an absence from these symptoms. Therefore, caution is warranted when interpreting the associations between metabolites and these two symptoms. Additionally, we examined symptom severity scores rather than using a binary presence/absence of these symptoms. This approach was chosen because severity scores may better capture symptom burden, differentiate levels of impairment, and serve as more informative, clinical indicators of treatment responsiveness or trajectories of symptom change than simple measures of symptom occurrence.

## Conclusion

In this study, we identified distinct metabolomic signatures associated with psychoneurological symptoms among breast cancer survivors, highlighting individual metabolites and broader metabolic pathways that may underpin symptom variability. Several metabolites exhibited robust associations with anxiety, fatigue, pain, and sleep disturbances, and the identification of compounds such as 5-hydroxyindoleacetylglycine and NA-Arg 18:3 across multiple symptom domains suggests shared biochemical pathways contributing to common symptom co-occurrence. Importantly, significant interactions between metabolites and race highlight the need to consider biological heterogeneity when examining symptoms, as metabolic processes may differentially influence PNS across racial groups. Pathway-level analyses further revealed that sulfur-containing amino acid metabolism, specifically taurine/hypotaurine and cysteine pathways, was among the most affected metabolic processes. These findings may provide a comprehensive view of the metabolic landscape associated with PNS in BCS, identifying potential biochemical contributors. Future work should prioritize longitudinal and mechanistic studies to validate these metabolic signatures across survivorship trajectories and explore whether targeted metabolomic or lifestyle interventions may alleviate symptom burden.

## Data Availability

The original contributions presented in the study are included in the article/**Supplementary Material**. Further inquiries can be directed to the corresponding author.
