# Supplementary material for: Serum metabolomic profiles associated with psychoneurological symptoms in women with early-stage breast cancer over one year
Source: Front Oncol. 2026 Mar 12;16:1779012. doi: 10.3389/fonc.2026.1779012 (PMC13019887; doi:10.3389/fonc.2026.1779012)
Supplement: Supplementary file 1 [file Table1.docx]

**Supplementary Table 1**

*Results of Statistically Significant Metabolites Associated with Anxiety, Sleep Disturbance, Fatigue, and Pain*

| **Symptom** | **Feature ID** | **Polarity** | **Coefficient** | ***p*-value** | **FDR *p*-value** |
| --- | --- | --- | --- | --- | --- |
| Anxiety | 238.0265-1.3 | Negative | 2.38 | 1.57 × 10^-7^ | 3.76 × 10^-4^ |
|  | 144.0437-2.39 | Negative | -2.40 | 5.79 × 10^-6^ | 6.87 × 10^-3^ |
|  | 100.0758-3.47 | Positive | -0.753 | 8.61 × 10^-6^ | 6.87 × 10^-3^ |
|  | 180.0206-2.4 | Negative | -2.07 | 2.83 × 10^-5^ | 1.42 × 10^-2^ |
|  | 230.976-2.89 | Negative | -1.66 | 2.96 × 10^-5^ | 1.42 × 10^-2^ |
|  | 197.0552-2.87 | Negative | -1.48 | 3.96 × 10^-5^ | 1.53 × 10^-2^ |
|  | 205.0732-2.13 | Negative | 1.21 | 4.47 × 10^-5^ | 1.53 × 10^-2^ |
|  | 232.9739-2.89 | Negative | -1.62 | 5.25 × 10^-5^ | 1.57 × 10^-2^ |
|  | 138.0058-3.48 | Negative | -0.961 | 6.40 × 10^-5^ | 1.58 × 10^-2^ |
|  | 211.0708-2.88 | Negative | -1.45 | 6.59 × 10^-5^ | 1.58 × 10^-2^ |
|  | 205.0732-1.99 | Negative | 1.15 | 1.08 × 10^-4^ | 2.32 × 10^-2^ |
|  | 155.0808-2.08 | Negative | 1.27 | 1.16 × 10^-4^ | 2.32 × 10^-2^ |
|  | 110.9564-1.56 | Negative | 1.54 | 1.36 × 10^-4^ | 2.51 × 10^-2^ |
|  | 187.0262-2.88 | Negative | -1.46 | 1.57 × 10^-4^ | 2.68 × 10^-2^ |
|  | 205.0732-1.91 | Negative | 1.13 | 1.70 × 10^-4^ | 2.71 × 10^-2^ |
|  | 163.9827-1.12 | Negative | -0.812 | 1.83 × 10^-4^ | 2.73 × 10^-2^ |
|  | 142.0856-1.88 | Negative | 1.03 | 2.17 × 10^-4^ | 3.06 × 10^-2^ |
|  | 169.0965-1.88 | Negative | 1.05 | 2.64 × 10^-4^ | 3.51 × 10^-2^ |
|  | 189.0233-2.89 | Negative | -1.73 | 3.52 × 10^-4^ | 4.44 × 10^-2^ |
| Sleep disturbance | 215.0365-1 | Negative | 8.62 | 3.58 × 10^-6^ | 2.88 × 10^-3^ |
|  | 221.0375-1.8 | Negative | 9.36 | 3.72 × 10^-6^ | 2.88 × 10^-3^ |
|  | 238.0265-1.3 | Negative | 13.6 | 4.23 × 10^-6^ | 2.88 × 10^-3^ |
|  | 205.0732-2.13 | Negative | 9.83 | 4.82 × 10^-6^ | 2.88 × 10^-3^ |
|  | 138.0058-3.48 | Negative | -6.47 | 1.15 × 10^-5^ | 5.49 × 10^-3^ |
|  | 205.0732-1.91 | Negative | 8.88 | 5.81 × 10^-5^ | 2.19 × 10^-2^ |
|  | 205.0732-1.99 | Negative | 8.82 | 6.39 × 10^-5^ | 2.19 × 10^-2^ |
|  | 256.1545-1.67 | Positive | 10.7 | 9.15 × 10^-5^ | 2.74 × 10^-2^ |
|  | 100.0758-3.47 | Positive | -4.26 | 1.27 × 10^-4^ | 3.39 × 10^-2^ |
|  | 142.0856-1.88 | Negative | 7.53 | 1.67 × 10^-4^ | 3.74 × 10^-2^ |
|  | 283.1543-1.4 | Positive | 6.40 | 1.76 × 10^-4^ | 3.74 × 10^-2^ |
|  | 169.0965-1.88 | Negative | 7.63 | 1.88 × 10^-4^ | 3.74 × 10^-2^ |
|  | 116.9569-1.07 | Negative | 7.02 | 2.44 × 10^-4^ | 4.10 × 10^-2^ |
|  | 300.181-1.4 | Positive | 6.79 | 2.56 × 10^-4^ | 4.10 × 10^-2^ |
|  | 239.0577-2.14 | Negative | 8.67 | 2.83 × 10^-4^ | 4.10 × 10^-2^ |
|  | 155.0808-2.08 | Negative | 8.70 | 3.04 × 10^-4^ | 4.10 × 10^-2^ |
|  | 265.1434-1.4 | Positive | 7.08 | 3.32 × 10^-4^ | 4.10 × 10^-2^ |
|  | 187.0525-1.7 | Negative | 6.77 | 3.87 × 10^-4^ | 4.10 × 10^-2^ |
|  | 83.0409-1.55 | Positive | 25.6 | 3.89 × 10^-4^ | 4.10 × 10^-2^ |
|  | 328.2123-1.37 | Positive | 6.86 | 3.96 × 10^-4^ | 4.10 × 10^-2^ |
|  | 209.1016-2.14 | Negative | 9.72 | 3.96 × 10^-4^ | 4.10 × 10^-2^ |
|  | 102.0288-3.38 | Negative | -4.39 | 4.07 × 10^-4^ | 4.10 × 10^-2^ |
|  | 96.0444-2.62 | Positive | 12.8 | 4.11 × 10^-4^ | 4.10 × 10^-2^ |
|  | 260.9404-1.35 | Negative | -5.54 | 4.11 × 10^-4^ | 4.10 × 10^-2^ |
|  | 820.738-1.19 | Positive | 9.83 | 5.19 × 10^-4^ | 4.57 × 10^-2^ |
|  | 155.0808-1.9 | Negative | 7.44 | 5.19 × 10^-4^ | 4.57 × 10^-2^ |
|  | 203.081-2.12 | Negative | 7.58 | 5.24 × 10^-4^ | 4.57 × 10^-2^ |
|  | 176.07-2.13 | Negative | 7.82 | 5.55 × 10^-4^ | 4.57 × 10^-2^ |
|  | 155.0808-2.29 | Negative | 9.05 | 5.56 × 10^-4^ | 4.57 × 10^-2^ |
|  | 321.1098-1.4 | Positive | 5.93 | 5.72 × 10^-4^ | 4.57 × 10^-2^ |
|  | 185.0269-1.51 | Negative | 5.92 | 6.20 × 10^-4^ | 4.71 × 10^-2^ |
|  | 127.0494-4.7 | Negative | 9.66 | 6.29 × 10^-4^ | 4.71 × 10^-2^ |
|  | 127.0495-4.52 | Negative | 9.70 | 6.78 × 10^-4^ | 4.92 × 10^-2^ |
|  | 180.9891-3.51 | Negative | -21.5 | 7.01 × 10^-4^ | 4.94 × 10^-2^ |
| Pain (Part B) | 556.315-6.96 | Negative | -2.40 | 1.22× 10^-6^ | 2.92 × 10^-3^ |
|  | 220.0801-4.24 | Negative | 1.30 | 1.29 × 10^-5^ | 1.55 × 10^-2^ |
|  | 220.08-4.42 | Negative | 1.26 | 2.26 × 10^-5^ | 1.80 × 10^-2^ |
|  | 341.1069-1.83 | Negative | -3.19 | 5.39 × 10^-5^ | 3.23 × 10^-2^ |
|  | 421.3176-1.33 | Positive | -0.502 | 9.73 × 10^-5^ | 4.66 × 10^-2^ |
| Fatigue (Part A) | 287.164-1.36 | Positive | 0.899 | 1.31 × 10^-5^ | 3.14 × 10^-2^ |
| Fatigue (Part B) | 148.0291-1.69 | Positive | -2.11 | 1.39 × 10^-9^ | 3.32 × 10^-6^ |
|  | 159.0838-4.05 | Negative | 1.12 | 5.03 × 10^-9^ | 6.02 × 10^-6^ |
|  | 144.0648-5.37 | Negative | 1.10 | 2.84 × 10^-8^ | 2.27 × 10^-5^ |
|  | 158.0805-4.04 | Negative | 1.02 | 3.79 × 10^-8^ | 2.27 × 10^-5^ |
|  | 158.0805-3.82 | Negative | 0.985 | 8.10 × 10^-8^ | 3.53 × 10^-5^ |
|  | 310.8968-3.51 | Negative | -1.87 | 8.85 × 10^-8^ | 3.53 × 10^-5^ |
|  | 194.0573-4.07 | Negative | 1.09 | 1.18 × 10^-7^ | 4.02 × 10^-5^ |
|  | 194.0573-4.23 | Negative | 1.02 | 5.50 × 10^-7^ | 1.52 × 10^-4^ |
|  | 251.0676-1.57 | Positive | -2.54 | 5.75 × 10^-7^ | 1.52 × 10^-4^ |
|  | 312.1596-1.34 | Positive | -0.837 | 6.33 × 10^-7^ | 1.52 × 10^-4^ |
|  | 209.0592-1.04 | Positive | -1.00 | 1.48 × 10^-6^ | 3.22 × 10^-4^ |
|  | 342.0503-1.74 | Positive | -4.16 | 2.10 × 10^-6^ | 4.08 × 10^-4^ |
|  | 211.0547-1.04 | Positive | -1.07 | 2.22 × 10^-6^ | 4.08 × 10^-4^ |
|  | 245.0143-1.44 | Negative | -0.773 | 2.99 × 10^-6^ | 5.12 × 10^-4^ |
|  | 329.1358-1.44 | Positive | 1.09 | 4.60 × 10^-6^ | 7.14 × 10^-4^ |
|  | 282.2278-1.6 | Positive | -0.793 | 4.77 × 10^-6^ | 7.14 × 10^-4^ |
|  | 564.3019-1.65 | Positive | -0.592 | 5.50 × 10^-6^ | 7.74 × 10^-4^ |
|  | 336.2383-1.43 | Positive | 1.49 | 8.83 × 10^-6^ | 1.18 × 10^-3^ |
|  | 164.1006-4.02 | Negative | 1.20 | 1.08 × 10^-5^ | 1.34 × 10^-3^ |
|  | 240.951-3.52 | Negative | -1.50 | 1.12 × 10^-5^ | 1.34 × 10^-3^ |
|  | 145.0681-5.36 | Negative | 1.31 | 1.28 × 10^-5^ | 1.46 × 10^-3^ |
|  | 286.144-1.35 | Positive | -0.622 | 1.67 × 10^-5^ | 1.82 × 10^-3^ |
|  | 421.3176-1.33 | Positive | -0.492 | 1.84 × 10^-5^ | 1.91 × 10^-3^ |
|  | 180.0415-5.37 | Negative | 1.40 | 2.21 × 10^-5^ | 2.17 × 10^-3^ |
|  | 174.9859-1.04 | Negative | -0.847 | 2.27 × 10^-5^ | 2.17 × 10^-3^ |
|  | 178.0493-2.69 | Negative | -0.944 | 2.46 × 10^-5^ | 2.26 × 10^-3^ |
|  | 172.9898-1.04 | Negative | -0.843 | 2.86 × 10^-5^ | 2.54 × 10^-3^ |
|  | 620.3646-1.62 | Positive | -1.21 | 3.42 × 10^-5^ | 2.93 × 10^-3^ |
|  | 313.1631-1.45 | Positive | 1.15 | 4.24 × 10^-5^ | 3.41 × 10^-3^ |
|  | 93.0322-1.04 | Negative | -0.854 | 4.27 × 10^-5^ | 3.41 × 10^-3^ |
|  | 146.0441-6.98 | Negative | 0.932 | 4.66 × 10^-5^ | 3.60 × 10^-3^ |
|  | 116.0335-7.01 | Negative | 0.927 | 5.89 × 10^-5^ | 4.41 × 10^-3^ |
|  | 164.1006-3.7 | Negative | 0.957 | 6.44 × 10^-5^ | 4.67 × 10^-3^ |
|  | 158.0805-3.59 | Negative | 0.610 | 6.75 × 10^-5^ | 4.75 × 10^-3^ |
|  | 585.2312-1.66 | Positive | -0.794 | 7.65 × 10^-5^ | 5.22 × 10^-3^ |
|  | 307.1384-1.46 | Negative | 1.19 | 7.85 × 10^-5^ | 5.22 × 10^-3^ |
|  | 226.9352-3.51 | Negative | -1.57 | 8.06 × 10^-5^ | 5.22 × 10^-3^ |
|  | 327.2136-1.45 | Positive | -1.06 | 1.32× 10^-4^ | 8.30 × 10^-3^ |
|  | 124.9896-6.13 | Negative | 1.46 | 1.92 × 10^-4^ | 1.18 × 10^-2^ |
|  | 437.3169-1.29 | Negative | -1.54 | 3.08 × 10^-4^ | 1.83 × 10^-2^ |
|  | 177.0916-1.97 | Positive | -0.937 | 3.13 × 10^-4^ | 1.83 × 10^-2^ |
|  | 254.9667-3.51 | Negative | -1.42 | 3.44× 10^-4^ | 1.96 × 10^-2^ |
|  | 152.0006-6.89 | Negative | 0.958 | 3.72 × 10^-4^ | 2.07 × 10^-2^ |
|  | 188.0913-3.56 | Negative | 0.608 | 4.26 × 10^-4^ | 2.32 × 10^-2^ |
|  | 298.0008-2.95 | Negative | -2.99 | 4.61 × 10^-4^ | 2.45 × 10^-2^ |
|  | 162.0542-1.43 | Negative | -0.902 | 5.14 × 10^-4^ | 2.67 × 10^-2^ |
|  | 250.164-1.79 | Positive | -1.44 | 5.25 × 10^-4^ | 2.67 × 10^-2^ |
|  | 269.2152-1.72 | Positive | -0.857 | 5.54 × 10^-4^ | 2.76 × 10^-2^ |
|  | _281.1749-1.39 | Positive | -1.07 | 6.06 × 10^-4^ | 2.96 × 10^-2^ |
|  | 298.2015-1.38 | Positive | -1.12 | 6.25 × 10^-4^ | 2.99 × 10^-2^ |
|  | 193.0685-4.98 | Negative | 1.07 | 6.69 × 10^-4^ | 3.14 × 10^-2^ |
|  | 321.153-1.47 | Negative | 1.01 | 6.90 × 10^-4^ | 3.18 × 10^-2^ |
|  | 165.0384-9.08 | Negative | 1.27 | 7.13 × 10^-4^ | 3.22 × 10^-2^ |
|  | 193.1436-1.61 | Positive | -1.25 | 7.56 × 10^-4^ | 3.28 × 10^-2^ |
|  | 192.0651-4.96 | Negative | 0.963 | 7.74 × 10^-4^ | 3.28 × 10^-2^ |
|  | 187.0258-3 | Negative | 0.546 | 7.76 × 10^-4^ | 3.28 × 10^-2^ |
|  | 192.0651-3.56 | Negative | 0.725 | 7.89 × 10^-4^ | 3.28 × 10^-2^ |
|  | 312.2375-1.78 | Positive | -0.785 | 7.95 × 10^-4^ | 3.28 × 10^-2^ |
|  | 261.0063-1.32 | Negative | 0.474 | 8.51 × 10^-4^ | 3.45 × 10^-2^ |
|  | 219.0764-1.69 | Positive | -2.94 | 9.79 × 10^-4^ | 3.73 × 10^-2^ |
|  | 633.4496-1.44 | Positive | -1.45 | 9.82 × 10^-4^ | 3.73 × 10^-2^ |
|  | 338.2171-1.46 | Positive | 0.763 | 1.00 × 10^-3^ | 3.73 × 10^-2^ |
|  | 180.0579-9.04 | Negative | 1.03 | 1.02 × 10^-3^ | 3.73 × 10^-2^ |
|  | 462.2735-1.56 | Positive | -1.29 | 1.02 × 10^-3^ | 3.73 × 10^-2^ |
|  | 213.0162-3.93 | Negative | 1.18 | 1.03 × 10^-3^ | 3.73 × 10^-2^ |
|  | 295.1212-1.66 | Negative | 1.10 | 1.03 × 10^-3^ | 3.73 × 10^-2^ |
|  | 126.0915-2.64 | Positive | 0.459 | 1.04 × 10^-3^ | 3.73 × 10^-2^ |
|  | 244.964-1.02 | Negative | 1.34 | 1.07 × 10^-3^ | 3.78 × 10^-2^ |
|  | 71.0121-9.03 | Negative | 1.10 | 1.10 × 10^-3^ | 3.82 × 10^-2^ |
|  | 322.9537-3.51 | Negative | -1.34 | 1.12 × 10^-3^ | 3.82 × 10^-2^ |
|  | 74.0716-7.71 | Positive | 1.38 | 1.17 × 10^-3^ | 3.95 × 10^-2^ |
|  | 217.9978-5.36 | Negative | 1.22 | 1.21 × 10^-3^ | 4.03 × 10^-2^ |
|  | 270.1702-1.62 | Positive | -0.811 | 1.23 × 10^-3^ | 4.03 × 10^-2^ |
|  | 294.1182-1.67 | Negative | 1.10 | 1.29 × 10^-3^ | 4.11 × 10^-2^ |
|  | 284-9834-1.04 | Negative | 0.934 | 1.29 × 10^-3^ | 4.11 × 10^-2^ |
|  | 326.2096-1.45 | Positive | -0.994 | 1.30 × 10^-3^ | 4.11 × 10^-2^ |
|  | 96.0444-2.62 | Positive | 1.31 | 1.37 × 10^-3^ | 4.25 × 10^-2^ |
|  | 269.0867-8.95 | Negative | 0.809 | 1.38 × 10^-3^ | 4.25 × 10^-2^ |
|  | 143.0331-9.03 | Negative | 1.05 | 1.53 × 10^-3^ | 4.65 × 10^-2^ |
|  | 149.985-6.16 | Negative | 0.574 | 1.64 × 10^-3^ | 4.90 × 10^-2^ |
|  | 49.044-9.01 | Negative | 1.08 | 1.66 × 10^-3^ | 4.91 × 10^-2^ |

***Note.*** A two-step approach was implemented for fatigue and pain due to their skewed distributions and the large number of zero numbers. In the first stage, the raw numeric scores were converted into a binary variable with two categories: 0 and greater than 0. Generalized estimating equations (GEE) with a logit link were then applied (Part A). In the second stage, the regression analysis focused on participants with non-zero GEE with Gaussian link for only the participants with non-zero scores (Part B).

FDR = false discovery rate.
