# Supplementary material for: Serum metabolomic profiles associated with psychoneurological symptoms in women with early-stage breast cancer over one year
Source: Front Oncol. 2026 Mar 12;16:1779012. doi: 10.3389/fonc.2026.1779012 (PMC13019887; doi:10.3389/fonc.2026.1779012)
Supplement: Supplementary file 2 [file Table2.docx]

**Supplementary Table 2**

*Results of Metabolite Associations and Their Interactions with Race*

| **Symptoms** | **Feature ID** | **Polarity** | **Coefficient** | ***p*-value** | **FDR *p*-value** |
| --- | --- | --- | --- | --- | --- |
| Depression | 250.2015-1.43 | Positive | 3.01 | 1.26 × 10^-5^ | 3.02 × 10^-2^ |
| Sleep disturbance | 126.0662-7.77 | Positive | 11.8 | 5.86 × 10^-8^ | 1.40 × 10^-4^ |
|  | 323.0878-1.67 | Negative | 9.80 | 8.29 × 10^-6^ | 9.93 × 10^-3^ |
|  | 245.1386-1.49 | Positive | -8.19 | 3.92 × 10^-5^ | 2.49 × 10^-2^ |
|  | 321.1098-1.4 | Positive | -10.5 | 4.16 × 10^-5^ | 2.49 × 10^-2^ |
| Pain (Part A) | 343.1153-1.62 | Positive | -1.69 | 1.30 × 10^-6^ | 3.11 × 10^-3^ |
| Pain (Part B) | 179.0557-2.11 | Negative | -0.722 | 1.91 × 10^-5^ | 1.73 × 10^-2^ |
|  | 275.1047-1.52 | Negative | -2.28 | 2.20 × 10^-5^ | 1.73 × 10^-2^ |
|  | 243.0863-1.65 | Negative | -1.77 | 2.58 × 10^-5^ | 1.73 × 10^-2^ |
|  | 148.0291-1.69 | Positive | 2.52 | 2.89 × 10^-5^ | 1.73 × 10^-2^ |
|  | 181.0118-3.37 | Negative | 0.614 | 4.49 × 10^-5^ | 1.92 × 10^-2^ |
|  | 491.2623-1.44 | Positive | -1.77 | 6.78 × 10^-5^ | 1.92 × 10^-2^ |
|  | 249.0872-1.3 | Positive | 7.33 | 6.91 × 10^-5^ | 1.92 × 10^-2^ |
|  | 844.6045-6.06 | Negative | -7.21 | 7.14 × 10^-5^ | 1.92 × 10^-2^ |
|  | 830.5869-6.06 | Negative | -7.74 | 7.22 × 10^-5^ | 1.92 × 10^-2^ |
|  | 172.0862-2.04 | Positive | -0.885 | 1.62 × 10^-4^ | 3.82 × 10^-2^ |
|  | 159.0899-3.71 | Positive | -1.69 | 1.76 × 10^-4^ | 3.82 × 10^-2^ |
|  | 183.0477-1.95 | Negative | 0.645 | 1.17 × 10^-4^ | 4.66 × 10^-2^ |
|  | 251.1277-1.5 | Negative | 0.756 | 2.17 × 10^-4^ | 4.08 × 10^-2^ |
|  | 145.035-3.36 | Negative | 0.651 | 2.69 × 10^-4^ | 4.08 × 10^-2^ |
|  | 154.0633-3.13 | Positive | -0.986 | 2.80 × 10^-4^ | 4.08 × 10^-2^ |
|  | 170.1178-1.45 | Positive | -1.90 | 2.87 × 10^-4^ | 4.08 × 10^-2^ |
|  | 147.0514-3.44 | Positive | 0.746 | 2.88 × 10^-4^ | 4.08 × 10^-2^ |
|  | 409.3672-1.32 | Negative | -1.45 | 2.90 × 10^-4^ | 4.08 × 10^-2^ |
|  | 139.0856-2.3 | Positive | -1.86 | 3.59 × 10^-4^ | 4.77 × 10^-2^ |
| Fatigue (Part B) | 830.5869-6.06 | Negative | -13.8 | 1.76 × 10^-8^ | 3.44 × 10^-5^ |
|  | 844.6045-6.06 | Negative | -12.9 | 2.87 × 10^-8^ | 3.44 × 10^-5^ |
|  | 249.0872-1.3 | Positive | 9.82 | 3.10 × 10^-7^ | 2.48 × 10^-4^ |
|  | 831.5909-6.06 | Negative | -11.8 | 8.94 × 10^-7^ | 5.35 × 10^-4^ |
|  | 148.0291-1.69 | Positive | 2.57 | 3.68 × 10^-6^ | 1.76 × 10^-3^ |
|  | 421.3176-1.33 | Positive | 3.94 | 1.03 × 10^-4^ | 4.11 × 10^-2^ |
|  | 251.0676-1.57 | Positive | 4.05 | 1.41 × 10^-4^ | 4.84 × 10^-2^ |

***Note.*** A two-step approach was implemented for fatigue and pain due to their skewed distributions and the large number of zero numbers. In the first stage, the raw numeric scores were converted into a binary variable with two categories: 0 and greater than 0. Generalized estimating equations (GEE) with a logit link were then applied (Part A). In the second stage, the regression analysis focused on the non-zero scores, using GEE with Gaussian link function (Part B).

The reference is the White race.

FDR = false discovery rate.
